# LC-MS/MS Screening of Phenolic Compounds in Wild and Cultivated Grapes *Vitis amurensis* Rupr.

**DOI:** 10.3390/molecules26123650

**Published:** 2021-06-15

**Authors:** Mayya Razgonova, Alexander Zakharenko, Konstantin Pikula, Yury Manakov, Sezai Ercisli, Irina Derbush, Evgeniy Kislin, Ivan Seryodkin, Andrey Sabitov, Tatiana Kalenik, Kirill Golokhvast

**Affiliations:** 1 N.I. Vavilov All-Russian Institute of Plant Genetic Resources, B. Morskaya 42-44, 190000 Saint-Petersburg, Russia; zakharenko.am@dvfu.ru (A.Z.); k.pikula@mail.ru (K.P.); labsad@bk.ru (I.D.); e.kislin@vir.nw.ru (E.K.); andrsabitov@rambler.ru (A.S.); k.golokhvast@vir.nw.ru (K.G.); 2 Far Eastern Federal University, 10 Ajax Bay, Russky Island, 690922 Vladivostok, Russia; kalenik.tk@dvfu.ru; 3 Siberian Federal Scientific Centre of Agrobiotechnology, Centralnaya, Presidium, 633501 Krasnoobsk, Russia; manakov@sfsca.ru; 4 Department of Horticulture, Agricultural Faculty, Ataturk University, 25240 Erzurum, Turkey; sercisli@atauni.edu.tr; 5 Pacific Geographical Institute, Far Eastern Branch of the Russian Academy of Sciences, Radio 7, 690041 Vladivostok, Russia; seryodkinivan@inbox.ru

**Keywords:** Amur grape, identification, mass spectrometry, metabolites, metabolomics

## Abstract

This work represents a comparative metabolomic study of extracts of wild grapes obtained from six different places in the Primorsky and Khabarovsk territories (Far East Russia) and extracts of grapes obtained from the collection of N.I. Vavilov All-Russian Institute of Plant Genetic Resources (St. Petersburg). The metabolome analysis was performed by liquid chromatography in combination with ion trap mass spectrometry. The results showed the presence of 118 compounds in ethanolic extracts of *V. amurensis* grapes. In addition, several metabolites were newly annotated in *V. amurensis*. The highest diversity of phenolic compounds was identified in the samples of the *V. amurensis* grape collected in the vicinity of Vyazemsky (Khabarovsk Territory) and the floodplain of the Arsenyevka River (Primorsky Territory), compared to the other wild samples and cultural grapes obtained in the collection of N.I. Vavilov All-Russian Institute of Plant Genetic Resources.

## 1. Introduction

The appearance of the first representatives of the *Vitaceae* family (genus *Vitis*) dates from the Upper Cretaceous period [1]. Several types of fossil grapes of genus *Vitis* have been found in different parts of North America [2]. In the Eocene, representatives of the genus *Vitis* were widespread in Eurasia and the Far North [2]. In the Paleogene, one of the best-preserved species of fossil grapes *Vitis sachalinensis* Krysht. was found and described in the sediments of the Sakhalin Island, the Russian Far East. These data show that the evolution of the vine in the territory of Russia proceeded from ancient times. Moreover, now wild grapes of the genus *Vitis* grow in many Russian regions [3,4]. At the same time, there is very little information about the culture of East Asian grapes.

Grape berries contain 65–85% water; 10–33% sugar (glucose and fructose); flofaben; gallic acid; quercetin; oenin; the glycosides monodelphinidin and delphinidin; the acids malic, hydrosilicic, *ortho*-hydroxybenzoic, phosphoric, tartaric, citric, succinic, formic, pectin, and tannins; salts of potassium; magnesium; calcium; manganese; cobalt; iron vitamins B1, B2, B6, B12, A, C, P, and PP; folic acid; and enzymes. The dominant class of biologically active compounds of fruits and especially grape ridges are flavonoids, in particular complexes of oligomeric proanthocyanidins (condensed tannins), which are polymeric forms of flavonoids from the group of catechins, and their monomeric units, namely catechins and leuсoanthocyanidins [5].

Many studies have been devoted to the biological activity of flavonoids and complexes of oligomeric proanthocyanidins [6,7]. Complexes of oligomeric proanthocyanidins act as traps of free radicals and block the process of lipid peroxidation of biological membranes [8,9]. Their antioxidant activity is many times higher than that of vitamins E and C. They can inhibit the activity of many enzymes (hydrolase, oxidoreductase, kinase, transferase, among others) [10]. Due to the wide spectrum of action, the active compounds of the grapes *V. amurensis* have a pronounced positive effect on various organs and systems of the body, such as antihypertensive and vasostrengthening effects, as well as antidiabetic, anti-inflammatory, antiallergy, anticarcinogenic, antistress, radioprotective, and antirheumatic effects. Moreover, flavonoids have an anti-Alzheimer’s activity [11,12,13].

This work presents a detailed comparative study of the metabolomic composition of wild *V. amurensis* grape berry extracts taken from six different locations of the Russian Far East and four cultural specimens of *V. amurensis* obtained from the collection of N.I. Vavilov All-Russian Institute of Plant Genetic Resources (St. Petersburg). High-performance liquid chromatography (HPLC) in combination with tandem mass spectrometry was used to identify target analytes in the extracts. Previously, the authors carried out metabolomic studies of Far Eastern plant species, such as *Schizandra chinensis*, *Rhodiola rosea*, *Rhododendron adamsii*, and *Panax ginseng* [14,15].

## 2. Results

The metabolome of ten samples of wild and cultural *V. amurensis* was analyzed and compared. A combination of both ionization modes (positive and negative) in MS full scan mode was applied for the molecular mass determination of the compounds in ethanolic extracts of *V. amurensis*. Compound identification was performed by comparing the observed *m*/*z* values and the fragmentation patterns with the literature. The list of compounds identified in the ethanolic extract of *V. amurensis* are represented in Table A1. The 118 compounds shown in Table A1 belong to different phenolic families, namely anthocyanidins, flavones, flavonols, flavan-3-ols, flavanones, hydroxycinnamic acids, hydroxybenzoic acids, stilbenes, and tannins.

### 2.1. Anthocyanidins and Anthocyanins

A total of 18 anthocyanin compounds have been identified in the analyzed samples of *V. amurensis* (Table 1). The anthocyanins pelargonidin-3-*O*-glucoside, cyanidin-3-*O*-glucoside, and petunidin-3-(6-*O*-coumaroyl) glucoside have already been characterized as a component of Far East *V. amurensis* [16]. The anthocyanins malvidin-3-*O*-acetylhexoside, delphinid-3,5-*O*-diglucoside, malvidin-3-*O*-rutinoside, malvidin 3-acetyl-5-glucoside, petunidin 3-coumaroylglucoside-5-*O*-glucoside, and malvidin 3-coumaroylglucoside-5-*O*-glucoside were only found in the extracts of cultivated *V. amurensis* (St. Petersburg).

### 2.2. Other Flavonoid Compounds

A total of 42 flavonoid compounds were identified in analyzed *V. amurensis* samples (Table 2). The flavonols dihydrokaempferol, kaempferide, mearnsetin, kaempferol-3-*O*-glucoside, dihydrokaempferol glucoside, isorhamnetin 3-*O*-rhamnoside, hyperoside, taxifolin-3-*O*-glucoside, kaempferol 3,7-di-*O*-glucoside, and quercetin-*O*-dihexoside have been already characterized as components of Far East *V. amurensis.*

### 2.3. Phenolic Acids and Other Compounds

In addition, 22 phenolic acids and 37 other compounds were identified in analyzed *V. amurensis* samples (Table 3). It should be noted that the coumarins umbelliferone and fraxin; the sterol fucosterol; and the flavanols taxifolin-3-O-glucoside, kaempferol-3,7-di-*O*-glucoside; hydroxycinnamic acids 3-*p*-coumaroyl-4-caffeoylquinic acid, and 5-*O*-(4’-*O*-*p*-coumaroyl glucosyl) quinic acid were identified by mass spectrometry only in samples of wild *V. amurensis* grapes collected from the Pakhtusov Islands and Rikord Island, Peter the Great Bay, Sea of Japan.

## 3. Discussion

In general, the diversity of phytochemicals identified in wild and cultural grape *V. amurensis* resulted in the following descending order (number of metabolites in parenthesis): VZK (52) > ART (46) > SPB-2 (39) > SPB-1 (28) > SPB-4 (27) > PAK (25) > RIK (22) > KAL (20) > SPB-3 (19) > ARS (18). The most diverse metabolome was identified in the grapes collected in the vicinity of Vyazemsky, Khabarovsk Territory, which was rich in flavanols and phenolic acids.

The anthocyanins identified in *V. amurensis* in this study were previously identified and annotated in the vines [17] *Solanium nigrum* [18], *Gaultheria Antarctica* [19], and *Vitis vinifera* [20] and wheat [21]. Our identification of flavonoid compounds agrees with bibliographic data for *Echinops* [22], *Rhodiola rosea* [23], *Ocimum* [24], *Alpinia officinarum* [25], Brazilian propolis [26], *Vitis vinifera* [20], *Rubus occidentalis* [27], *C. edulis* [28], and *Vaccinium macrocarpon* [29].

Although wild grapes tend to be more diverse than cultivated varieties [30], this number of anthocyanins in one form is quite rare and more likely to occur in other berries, such as blueberries [31]. We hypothesize that many different anthocyanins are associated with rather low temperatures in summer and monsoon climates. To respond to adverse conditions, various anthocyanins are produced [32]. In addition, *V. amurensis* have an increased acidity of the fruit, which is also associated with unfavorable growing conditions [33]. As it is known, anthocyanins and many other phenolic compounds participating in the protective processes of plants are more stable in an acidic environment [34].

## 4. Materials and Methods

### 4.1. V. amurensis Samples

**Figure 1:**
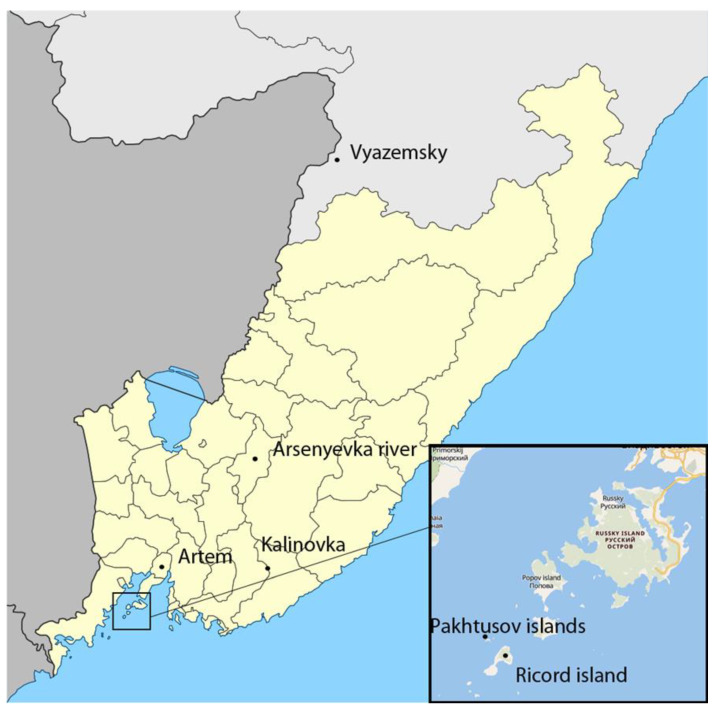
Region of wild *V. amurensis* grape collection.

Ten samples of wild and cultivated grape *V. amurensis* were selected for the performance of metabolomic study. Six samples of wild *V. amurensis* were collected from different places in the Primorsky and Khabarovsk territories, Far Eastern Russia (Table 4, Figure 1). Four samples of cultivated *V. amurensis*, namely SPB-1, SPB-2, SPB-3, and SPB-4, were obtained from the collection of N.I. Vavilov All-Russian Institute of Plant Genetic Resources, St. Petersburg. The grapes were harvested at the end of August and September 2020. Each sample included 100 g of grape berries.

### 4.2. Chemicals and Reagents

HPLC-grade acetonitrile was purchased from Fisher Scientific (Southborough, UK), and MS-grade formic acid was purchased from Sigma-Aldrich (Steinheim, Germany). Ultra-pure water was obtained with Siemens Ultra-Clear TWF EDI UV UF TM Water Purification System (Siemens, Munich, Germany). All the other chemicals were of analytical grade.

### 4.3. Fractional Maceration

Fractional maceration with ethyl alcohol was applied to obtain highly concentrated extracts of *V. amurensis*. Each sample of *V. amurensis* was divided into three parts and consistently infused. The infusion time of each part of the extractant was seven days.

### 4.4. Liquid Chromatography

The separation of multicomponent mixtures was performed by a Shimadzu LC-20 Prominence HPLC (Shimadzu, Kyoto, Japan) equipped with a UV detector and a Shodex ODP-40 4E reverse-phase column (4.6 × 250 mm, particle size 4 µm). The gradient elution program with two mobile phases (A, deionized water; B, acetonitrile with formic acid 0.1% *v*/*v*) was as follows: 0.01–2 min, 100% B; 2–50 min, 100–0% B; control washing 50–60 min, 0% B. The entire HPLC analysis was done with an SPD-20A detector at wavelengths of 230 and 330 nm; the temperature corresponded to 40 °C. The injection volume was 10 µL.

### 4.5. Mass Spectrometry

MS analysis was performed on an ion trap amaZon SL (Bruker Daltonics, Bremen, Germany). Four-stage ion separation (MS/MS mode) was implemented. All the chemical profiles of the samples were obtained by the HPLC–ESI–MS/MS method. The working parameters were as follows: ionization source temperature 50 °C, gas flow 4 L/min, nebulizer gas (atomizer) 7.3 psi, capillary voltage 4500 V, endplate bend voltage 1500 V, fragmentary voltage 280 V, and collision energy 60 eV. The ion trap was used in the scan range of 100–1.700 *m*/*z* for MS and MS/MS. The capture rate was one spectrum/s for MS and two spectrum/s for MS/MS. The mass spectra were recorded in negative and positive ion mode. Data collection was controlled by Hystar DataAnalisys 4.1 software (Bruker Daltonics, Bremen, Germany). All the measurements were performed in triplicate.

## Figures and Tables

**Table 1:** Anthocyanins identified in the ethanolic extracts of *V. amurensis*.

No.	Identified Compound	ARS	ART	KAL	PAK	RIK	VZK	SPB-1	SPB-2	SPB-3	SPB-4
1.	Cyanidin 3,5-*O*-diglucoside			+			+	+	+	+	
2.	Cyanidin-3-*O*-glucoside [Cyanidin 3-*O*-beta-*D*-glucoside]			+							
3.	Delphinidin 3-*O*-glucoside		+						+		
4.	Delphinidin-3,5-*O*-diglucoside							+			
5.	Malvidin 3-(6-*O*-acetyl) glucoside	+	+								+
6.	Malvidin 3-(6-*O*-coumaroyl) glucoside		+								+
7.	Malvidin 3-(6’-p-caffeoylglucoside)	+	+	+			+		+	+	
8.	Malvidin 3,5-diglucoside		+		+	+	+	+	+	+	+
9.	Malvidin 3-coumaroylglucoside-5-*O*-glucoside										+
10.	Malvidin 3-*O*-acetyl hexoside							+			
11.	Malvidin 3-*O*-glucoside		+	+		+	+	+	+	+	+
12.	Pelargonidin-3-*O*-glucoside (callistephin)						+				
13.	Peonidin-3,5-*О*-diglucoside [peonin; peonidin 3-glucoside-5-glucoside]		+			+	+	+	+	+	
14.	Peonidin-3-*O*-glucoside						+	+	+		
15.	Petunidin 3-(6-*O*-coumaroyl) glucoside		+								
16.	Petunidin 3-coumaroylglucoside-5-*O*-glucoside										+
17.	Petunidin 3-*O*-glucoside-5-*O*-glucoside [Petunidin 3,5-di-*O*-beta-*D*-glucoside]		+	+			+		+	+	
18.	Petunidin-3-*O*-glucoside		+								
	Total number	2	10	5	1	3	8	7	8	6	6

ARS, wild *V. amurensis* sample obtained from floodplain of the Arsenyevka River (Primorsky Territory); ART, wild *V. amurensis* sample obtained from the vicinity of Artem (Primorsky Territory); KAL, wild *V. amurensis* sample obtained from the vicinity of Kalinovka (Primorsky Territory); PAK, wild *V. amurensis* sample obtained from the Pakhtusov Islands (Sea of Japan); RIK, wild *V. amurensis* sample obtained from Rikord Island (Sea of Japan); VZK, wild *V. amurensis* sample obtained from the vicinity of Vyazemsky (Khabarovsk Territory); SPB-1, SPB-2, SPB-3, and SPB-4, samples of cultivated *V. amurensis* provided by N.I. Vavilov All-Russian Institute of Plant Genetic Resources (St. Petersburg).

**Table 2:** Other flavonoid compounds identified in the ethanolic extracts of *V. amurensis*.

No.	Identified Compound	ARS	ART	KAL	PAK		RIK		VZK			SPB-1			SPB-2		SPB-3		SPB-4	
	Flavonols																			
1.	Quercetin-3-*O*-glucuronide	+	+	+	+		+		+						+		+		+	
2.	Kaempferol	+	+	+			+		+						+					
3.	Quercetin			+			+		+			+							+	
4.	Isorhamnetin [Isorhamnetol; Quercetin 3’-Methyl ether]		+									+			+				+	
5.	Isorhamnetin 3-*O*-glucoside								+			+			+		+			
6.	Myricetin-3-*O*-galactoside		+						+						+		+			
7.	Quercetin 3-*O*-glucoside [Isoquercitrin; Hirsutrin]		+						+						+				+	
8.	Myricetin		+	+											+					
9.	Dihydrokaempferol		+						+											
10.	Dihydroquercetin (Taxifolin; Taxifoliol)						+												+	
11.	Hyperoside (Quercetin 3-*O*-galactoside; Hyperin)	+							+											
12.	Kaempferol diglycoside				+		+													
13.	Kaempferol glycoside	+							+											
14.	Dihydrokaempferol glucoside	+																		
15.	Herbacetin														+					
16.	Isorhamnetin 3-*O*-rhamonoside								+											
17.	Kaempferide		+																	
18.	Mearnsetin		+																	
19.	Quercetin-*O*-dihexoside		+																	
20.	Rutin (Quercetin 3-*O*-rutinoside)		+																	
21.	Taxifolin-3-*O*-glucoside						+													
	Total number:	3	9	2	1		4		8			3			6		2		4	
	Flavones																			
22.	Apigenin	+	+	+	+				+						+					
23.	Syringetin			+								+			+		+			
24.	Luteolin diglycoside											+			+		+			
25.	Nevadensin		+				+													
26.	Vitexin 2”-*O*-glucoside [Apigenin 8-*C*-glucoside 2”-*O*-glucoside]		+																+	
27.	Luteolin				+															
28.	Diosmetin [Luteolin 4’-Methyl Ether; Salinigricoflavonol]																		+	
29.	Pentahydroxy trimethoxy flavone																		+	
30.	Apigenin diglycoside								+											
31.	Vitexin [ Apigenin 8-*C*-Glucoside]														+					
32.	Vitexin glucoside	+																		
33.	Apigenin glucoside								+											
	Total number:	2	3	2	2		1		3			2			4		2		3	
	Dimethoxyflavone																			
34.	Cirsimaritin [Scrophulein; 4’,5-dihydroxy-6,7-dimethoxyflavone; 7-methylcapillarisin]		+																	
	Flavan-3-ols																			
35.	Catechin [D-Catechol]		+		+		+		+			+			+		+		+	
36.	Epicatechin		+		+															
37.	Gallocatechin [+(-)Gallocatechin]														+					
38.	Catechin gallate								+											
	Total number:	0	2	0	2		1		2			1			2		1		1	
	Flavanones																			
39.	Naringenin [Naringetol; Naringenine]				+		+		+											
40.	Eriodictyol-7-*O*-glucoside [Pyracanthoside; Miscanthoside]				+														+	
41.	Hesperitin [Hesperetin]								+											
42.	Hexahydroxyflavanone hexoside		+																	
	Total number:	0	1	0	2		1		2			0			0		0		1	

ARS, wild *V. amurensis* sample obtained from floodplain of the Arsenyevka River (Primorsky Territory); ART, wild *V. amurensis* sample obtained from the vicinity of Artem (Primorsky Territory); KAL, wild *V. amurensis* sample obtained from the vicinity of Kalinovka (Primorsky Territory); PAK, wild *V. amurensis* sample obtained from the Pakhtusov Islands (Sea of Japan); RIK, wild *V. amurensis* sample obtained from Rikord Island (Sea of Japan); VZK, wild *V. amurensis* sample obtained from the vicinity of Vyazemsky (Khabarovsk Territory); SPB-1, SPB-2, SPB-3, and SPB-4, samples of cultivated *V. amurensis* provided by N.I. Vavilov All-Russian Institute of Plant Genetic Resources (St. Petersburg).

**Table 3:** Phenolic acids and other compounds identified in the ethanolic extracts of *V. amurensis*.

No.	Identified Compound	ARS	ART	KAL	PAK	RIK	VZK	SPB-1	SPB-2	SPB-3	SPB-4
	Hydroxybenzoic acids										
1.	Salvianolic acid D		+		+		+		+		+
2.	Salvianolic acid G	+					+		+		
3.	Ellagic acid [Benzoaric acid; Elagostasine]						+			+	
4.	4-Hydroxybenzoic acid							+			
5.	Protocatechuic acid							+			
6.	Gallic acid						+				
7.	Syringic acid [Benzoic acid; Cedar acid]								+		
8.	Salvianolic acid F						+				
9.	Dihydroxybenzoyl-hexoside						+				
	Total number:	1	1	0	1	0	6	2	3	1	1
	Hydroxycinnamic acids										
10.	Caftaric acid [cis-caftaric acid; 2-caffeoyl-L-tartaric acid; caffeoyl tartaric acid}	+		+	+	+	+		+	+	+
11.	Di-*O*-caffeoylquinic acid		+					+	+		
12.	Sinapic acid [trans-Sinapic acid]			+			+				
13.	Coutaric acid [Trans-*p*-Coumaroyltartaric acid]					+					+
14.	Fertaric acid [Fertarate]				+						+
15.	p-Coumaric acid-O-hexoside [Trans-*p*-Coumaric acid 4-glucoside]						+				+
16.	Caffeic acid-*O*-(sinapoyl-*O*-hexoside)							+	+		
17.	*p*-Coumaric acid				+						
18.	Caffeoylmalic acid		+								
19.	1-Caffeoyl-beta-*D*-glucose [Caffeic acid-glucoside]										+
20.	5-*O*-(4’-*O*-*p*-coumaroyl glucosyl) quinic acid				+						
21.	3-*p*-coumaroyl-4-caffeoylquinic acid					+					
22.	Coumaric acid derivative						+				
	Total number:	0	1	0	3	2	2	1	1	0	4
	Other compounds										
23.	Ethyl gallate	+	+	+	+		+	+	+	+	+
24.	Malic acid	+	+		+	+	+	+	+		
25.	Hexose-hexose-N-acetyl	+	+				+	+	+	+	
26.	Citric acid		+	+			+	+	+		
27.	Quinic acid		+		+	+	+	+			
28.	Galloyl glucose [Beta-Glucogallin; 1-*O*-Galloyl-Beta-D-Glucose]		+	+		+			+		+
29.	L-Tryptophan [Tryptophan; (S)-Tryptophan]		+	+				+	+		
30.	Cyclopassifloic acid glucoside	+	+			+	+				
31.	Indole-3-carboxylic acid		+		+	+					
32.	Myristoleic acid [Cis-9-Tetradecanoic acid]		+				+		+		
33.	Resveratrol [trans-Resveratrol; Stilbentriol]	+	+	+							
34.	Protocatechuic acid-*O*-hexoside				+		+			+	
35.	Palmatine [Berbericinine; Burasaine]						+	+			+
36.	Polydatin [Piceid; trans-Piceid]				+		+		+		
37.	Procyanidin A-type dimer		+					+	+		
38.	Shikimic acid		+					+			
39.	Esculetin [Cichorigenin; Aesculetin]				+		+				
40.	9-oxo-10E,12Z-octadecanoic acid [9-Oxo-ODE]				+					+	
41.	Gallic acid hexoside				+				+		
42.	Esculin [Aesculin; Esculoside; Polichrome]			+		+					
43.	1-*O*-Sinapoyl-beta-D-glucose			+						+	
44.	Stigmasterol [Stigmasterin; Beta-Stigmasterol]	+							+		
45.	Oleanoic acid		+				+				
46.	Tartaric acid						+				
47.	Umbelliferone				+						
48.	Dihydroferulic acid						+				
49.	Linolenic acid (Alpha-Linolenic acid; Linolenate)						+				
50.	Nonadecadienoic acid							+			
51.	Bilobalide [ (-)-Bilobalide]		+								
52.	3,7 -Dimethylquercetin										+
53.	Erucic acid (Cis-13-Docosenoic acid)							+			
54.	Fraxin (Fraxetin-8-*O*-glucoside)					+					
55.	Fucosterol [Fucostein; Trans-24-Ethylidenecholesterol]				+						
56.	Phlorizin [Phloridzin; Phlorizoside; Floridzin: phlorrhizin; Phloretin 2’-Glucoside; Phloretin-O-hexoside]	+									
57.	Ursolic acid						+				
58.	Anmurcoic acid										+
59.	Dimethylellagic acid hexose						+				
	Total number	7	15	7	11	7	17	11	11	5	5

ARS, wild *V. amurensis* sample obtained from floodplain of the Arsenyevka River (Primorsky Territory); ART, wild *V. amurensis* sample obtained from the vicinity of Artem (Primorsky Territory); KAL, wild *V. amurensis* sample obtained from the vicinity of Kalinovka (Primorsky Territory); PAK, wild *V. amurensis* sample obtained from the Pakhtusov Islands (Sea of Japan); RIK, wild *V. amurensis* sample obtained from Rikord Island (Sea of Japan); VZK, wild *V. amurensis* sample obtained from the vicinity of Vyazemsky (Khabarovsk Territory); SPB-1, SPB-2, SPB-3, and SPB-4, samples of cultivated *V. amurensis* provided by N.I. Vavilov All-Russian Institute of Plant Genetic Resources (St. Petersburg).

**Table 4:** Locations of wild *V. amurensis* grape collection.

Code Name of the Sample	Location	Geographical Values	Soil Type
ARS	Floodplain of the Arsenyevka River, Primorsky Territory	N. 44°52′18″, E 133°35′12″	brown grey bleached soils
ART	The vicinity of Artem, Primorsky Territory	N 43°21′34″, E 132°11′19″	yellow-brown soil
KAL	The vicinity of Kalinovka, Primorsky Territory	N 43°07′27″, E 133°12′30″	layered floodplains
PAK	The Pakhtusov Islands, Peter the Great Bay, Sea of Japan	N 42°53′57″, E 131°38′45″	yellow-brown soil
RIK	Rikord Island, Peter the Great Bay, Sea of Japan	N 42°52′54″, E 131°40′06″	yellow-brown earth soils
VZK	The vicinity of Vyazemsky, Khabarovsk Territory	N 47°32′15″, E 134°45′20″	podzolic brown forest heavy loamy soils

## Data Availability

Not applicable.

## Appendix A

**Table A1:** The list of compounds identified in ethanolic extracts of *V. amurensis*.

No.	Identified Compound	Molecular Formula	Calculated Mass	Precursor Ion, *m*/*z*		Fragment Ions, *m*/*z*	References
				[M–H]^–^	[M+H]^+^		
	Anthocyanins						
1.	Cyanidin 3,5-*O*-diglucoside	C_27_H_31_O_16_	611.5335		611	287; 449; 269; 231; 199; 161; 231; 213; 189; 175; 147	[35,36]
2.	Cyanidin-3-*O*-glucoside	C_21_H_21_O_11_	449.3848		449	287; 206; 143	[19,20,35,37,38]
3.	Delphinidin 3-*O*-glucoside	C_21_H_21_O_12+_	465.3905		465	303; 257; 229; 201; 165; 239; 213; 173; 145; 117	[19,20,21,39]
4.	Delphinidin-3,5-*O*-diglucoside	C_27_H_30_O_17_	626.5169		627	465; 303; 257; 153; 229; 155	[18,40]
5.	Malvidin 3,5-*O*-diglucoside	C_32_H_31_O_15_	655.5795		655	493; 331; 315; 179; 313	[17,20,21]
6.	Malvidin 3-(6-*O*-acetyl) glucoside	C_25_H_27_O_13_	535.478		537	331; 299; 261; 243; 211; 154; 111	[20,39]
7.	Malvidin 3-(6-*O*-coumaroyl) glucoside	C_32_H_31_O_14_	639.5801		639	331; 315; 299; 270; 242; 179; 150; 287; 213	[20,39,40]
8.	Malvidin 3-coumaroylglucoside-5-*O*-glucoside	C_35_H_45_O_21_	801.7192		801	639; 493; 331; 315; 287; 270; 242; 300	[39]
9.	Malvidin 3-*O*-acetyl hexoside	C_25_H_27_O_14_	535.479		537	331; 305; 261; 207; 185; 255; 229; 211	[17]
10.	Malvidin 3-*O*-glucoside	C_23_H_25_O_12_	493.4374		493	331; 315; 179	[20,39,40]
11.	Pelargonidin-3-*O*-glucoside (callistephin)	C_21_H_21_O_10_	433.3854		433	414; 271; 172; 226; 116	[35,39,41]
12.	Peonidin-3,5-*О*-diglucoside [Peonin; Peonidin 3-glucoside-5-glucoside]	C_28_H_33_O_16_	625.5520		625	301; 463; 286; 258	[21,39,40]
13.	Peonidin-3-*O*-glucoside	C_22_H_23_O_11 +_	463.4114		463	301; 286; 268; 258; 230; 202; 174; 121	[20,39,41]
14.	Petunidin 3-(6-*O*-coumaroyl) glucoside	C_31_H_29_O_14_	625.553		625	317; 302; 274; 218	[20,39,40]
15.	Petunidin 3-coumaroylglucoside-5-*O*-glucoside	C_34_H_43_O_21_	787.6926		787	625; 479; 317; 301; 246; 302; 274; 228	[39,40]
16.	Petunidin 3-galactoside	C_22_H_23_O_12+_	479.4108		479	317; 302; 273	[19,20,21,39]
17.	Petunidin 3,5-diglucoside	C_28_H_33_O_17_	641.5514		641	317; 479; 420; 257; 302; 274; 228	[39,40]
	Flavonols						
18.	Dihydrokaempferol	C_15_H_12_O_6_	288.2522		289	271; 199; 127; 243; 189; 118	[22,42]
19.	Dihydrokaempferol glucoside	C_21_H_22_O_11_	450.3928	449		287; 227; 269; 225; 149	[27]
20.	Dihydroquercetin (taxifolin; taxifoliol)	C_15_H_12_O_7_	304.2516		305	259; 149; 199; 241; 159; 171	[20,43,44]
21.	Herbacetin [3,5,7,8-tetrahydroxy-2-(4-hydro- xyphenyl)-4H-chromen-4-one]	C_15_H_10_O_7_	302.2357	301		179; 273; 121; 151	[24,45]
22.	Hyperoside (quercetin 3-*O*-galactoside; hyperin)	C_21_H_20_O_12_	464.3763	463		301; 179; 257; 255; 147	[43,46,47,48]
23.	Isorhamnetin [isorhamnetol; quercetin 3’-methyl ether; 3-methylquercetin]	C_16_H_12_O_7_	316.2623		317	299; 270; 230; 207;177; 165;147; 123; 147; 123; 119	[49,50]
24.	Isorhamnetin 3-*O*-glucoside	C_22_H_22_O_12_	478.4029		479	317; 301; 257; 274; 228; 150	[20,47,51]
25.	Isorhamnetin 3-*O*-rhamonoside	C_22_H_22_O_11_	462.4035	461		315; 152; 219	[28,49]
26.	Kaempferide	C_16_H_12_O_6_	300.2629		301	283; 265; 239; 211; 185; 133; 151	[20,24,26]
27.	Kaempferol	C_15_H_10_O_6_	286.2363		287	269; 227; 153	[20,24,50]
28.	Kaempferol diglycoside	C_27_H_30_O_16_	610.5175		611	449; 287; 229; 165; 213; 111	[52,53]
29.	Kaempferol glycoside	C_21_H_20_O_11_	448.3769		449	287; 269; 217	[20,47]
30.	Mearnsetin	C_16_H_12_O_8_	332.2617		333	318; 301; 273; 245; 193; 165; 139; 289; 271; 219; 153; 136	[49]
31.	Myricetin	C_15_H_10_O_8_	318.2351	317		273; 191; 255; 229; 205; 187; 163; 125; 227	[20,28,54]
32.	Myricetin-3-*O*-galactoside	C_21_H_20_O_13_	480.3757	479		299; 153; 271; 243; 171	[47,48,55]
33.	Quercetin	C_15_H_10_O_7_	302.2357		303	285; 163; 267; 159; 239	[20,24,37,43]
34.	Quercetin 3-*O*-glucoside [Isoquercitrin; Hirsutrin]	C_21_H_20_O_12_	464.3763		465	303; 285; 257; 229; 201; 150; 155	[20,27,47,56]
35.	Quercetin-3-*O*-glucuronide	C_21_H_18_O_13_	478.3598	477		301; 179; 273; 151	[39,47,57]
36.	Quercetin-*O*-dihexoside	C_27_H_30_O_17_	626.5179		627	303; 257; 150; 229	[51,58]
37.	Rutin (quercetin 3-*O*-rutinoside)	C_27_H_30_O_16_	610.5175		611	303; 229; 257	[27,35,37,56]
38.	Taxifolin-3-*O*-glucoside	C_21_H_22_O_12_	466.3922		467	449; 303; 188; 287; 132; 260	[20]
	Flavones						
39.	Apigenin [5,7-dixydroxy-2-(40hydroxyphenyl)-4H-chromen-4-one]	C_15_H_10_O_5_	270.2369		271	253; 181; 137	[56,59,60]
40.	Luteolin	C_15_H_10_O_6_	286.2363		287	271; 225; 175; 158	[43,56,59,60]
41.	Diosmetin [luteolin 4’-methyl ether; salinigricoflavonol]	C_16_H_12_O_6_	300.2629		301	286; 258; 229; 184; 153; 124	[61,62,63]
42.	Cirsimaritin [scrophulein; 4’,5-dihydroxy-6,7-dimethoxyflavone; 7-methylcapillarisin]	C_17_H_14_O_6_	314.2895	313		298; 247; 151; 270	[24]
43.	Nevadensin	C_18_H_16_O_7_	344.3154	343		328; 259; 313; 269	[24,63]
44.	Syringetin	C_17_H_14_O_8_	346.2883	345		330; 315; 246; 151; 287; 271; 203; 183; 163	[28]
45.	Pentahydroxy trimethoxy flavone	C_18_H_16_O_10_	392.3136		393	378; 347; 317; 284; 246; 206; 349; 321; 284; 193; 322; 304;282; 196; 154	[28]
46.	Apigenin diglycoside	C_21_H_20_O_10_	432.3775		433	414; 287; 186; 241; 158	[20,56,64,65]
47.	Vitexin [apigenin 8-C-glucoside]	C_21_H_20_O_10_	432.3775	431		249; 221; 192	[57,66,67]
48.	Luteolin diglycoside	C_21_H_20_O_11_	448.3769		449	287; 213; 137; 185	[20,55,56,66,68]
49.	Isovitexin 6”-*O*-deoxyhexoside [apigenin 6-C-glucoside 6”-*O*-deoxyhexoside]	C_27_H_30_O_14_	578.5187		579	415; 297; 177; 397; 344; 362	[66]
50.	Vitexin glucoside	C_27_H_30_O_15_	594.5181		595	415; 353; 283; 265; 176	[66]
51.	Apigenin glucoside	C_29_H_32_O_15_	620.5554		621	561; 547; 461; 533; 461; 433	[66]
	Flavan-3-ols						
52.	Catechin [D-catechol]	C_15_H_14_O_6_	290.2681	289		245; 205; 203; 188	[43,49,55,57]
53.	Epicatechin	C_15_H_14_O_6_	290.2681		291	272; 175; 130; 157; 140	[20,49,55]
54.	Gallocatechin [+(-)gallocatechin]	C_15_H_14_O_7_	306.2675	305		179; 125	[20,28,43,44]
55.	Catechin gallate	C_22_H_18_O_10_	442.3723	441		289; 169; 245; 205; 203	[20,56]
	Flavanones						
56.	Naringenin [Naringetol; Naringenine]	C_15_H_12_O_5_	272.5228		273	227; 155; 209; 139	[20,43,49]
57.	Hesperitin [Hesperetin]	C_16_H_14_O_6_	302.2788	301		257; 151; 228; 189	[20,43,68]
58.	Eriodictyol-7-*O*-glucoside [Pyracanthoside; miscanthoside]	C_21_H_22_O_11_	450.3928	449		269; 207; 251; 165	[48,65,68]
59.	Hexahydroxyflavanone hexoside	C_21_H_22_O_13_	482.3916		483	437; 359; 263; 231; 298; 255; 225; 155	[28]
	Hydroxybenzoic acids						
60.	4-hydroxybenzoic acid	C_7_H_6_O_3_	138.1207		139	121	[20,69,70]
61.	Protocatechuic acid	C_7_H_6_O_4_	154.1201		155	127	[20,28,55]
62.	Gallic acid	C_7_H_6_O_5_	170.1195		171	126	[20,54,55]
63.	Syringic acid [benzoic acid; cedar acid]	C_9_H_10_O_5_	198.1727		199	154; 140; 111; 140; 123; 125	[20,55,71]
64.	Ellagic acid [benzoaric acid; elagostasine]	C_14_H_6_O_8_	302.1926		303	172; 158; 144; 127; 116	[27,41,44]
65.	Salvianolic acid F	C_17_H_14_O_6_	314.2895		315	269; 243; 213;185; 144; 207; 181; 153; 179; 161; 133	[69]
66.	Dihydroxybenzoyl-hexoside	C_13_H_16_O_9_	316.2607	315		153; 253; 151; 184	[66]
67.	Salvianolic acid G	C_18_H_12_O_7_	340.2837		341	323; 295; 255; 195; 159; 305	[63,72]
68.	Salvianolic acid D	C_20_H_18_O_10_	418.3509	417		373; 329; 287; 209	[69,73]
	Hydroxycinnamic acids						
69.	*p*-Coumaric acid	C_9_H_8_O_3_	164.16		165	146; 119	[20,46,55,73]
70.	Sinapic acid [trans-sinapic acid]	C_11_H_12_O_5_	224.2100		225	179; 153; 115; 133; 115	[20,37,55,74]
71.	Caffeoylmalic acid	C_13_H_12_O_8_	296.2296	295		133; 179; 148; 119; 115	[28]
72.	Coutaric acid [trans-*p*-Coumaroyltartaric acid]	C_13_H_12_O_8_	296.2296	295		163; 119	[20]
73.	Caftaric acid [cis-caftaric acid; 2-caffeoyl-L-tartaric acid; caffeoyl tartaric acid}	C_13_H_12_O_9_	312.23	311		149; 221; 131	[20,38,64,69]
74.	Fertaric acid [fertarate]	C_14_H_14_O_9_	326.2556	325		193; 149; 134	[20]
75.	p-Coumaric acid-*O*-hexoside [trans-*p*-coumaric acid 4-glucoside]	C_15_H_18_O_8_	326.2986	325		193; 163; 119	[28,57,75]
76.	1-caffeoyl-beta-D-glucose [caffeic acid-glucoside]	C_15_H_18_O_9_	342.298	341		179; 161; 135	[20,66]
77.	5-*O*-(4’-*O*-*p*-coumaroyl glucosyl) quinic acid	C_22_H_28_O_13_	500.4499		501	339; 277; 203	[56]
78.	3-*p*-coumaroyl-4-caffeoylquinic acid	C_25_H_24_O_11_	500.4515		501	355; 483; 181; 225; 281; 193; 120; 133	[76]
79.	Coumaric acid derivative	C_30_H_30_O_7_	502.5550		503	457; 411; 382; 339; 293; 409; 391; 367; 323; 293; 233; 205	[57]
80.	Di-*O*-caffeoylquinic acid	C_25_H_24_O_12_	516.4509		517	355; 339; 202	[58,66,76]
81.	Caffeic acid-*O*-(sinapoyl-*O*-hexoside)	C_26_H_30_O_14_	566.5080		567	405; 520; 249; 234	[57,77]
	Other compounds						
82.	Malic acid	C_4_H_6_O_5_	134.0874	133		115	[57,69,78]
83.	Tartaric acid	C_4_H_6_O_6_	150.0900	149		131	[78,79]
84.	Umbelliferone	C_9_H_6_O_3_	162.1421	161		115	[20,28,54]
85.	Shikimic acid	C_7_H_10_O_5_	174.1513		175	112	[28,78]
86.	Indole-3-carboxylic acid	C_10_H_9_NO_2_	175.1840		176	130	[75]
87.	Esculetin [Cichorigenin; Aesculetin]	C_9_H_6_O_4_	178.1415		179	133; 115	[20]
88.	Citric acid	C_6_H_8_O_7_	192.1235	191		111; 173; 143; 127	[57,59,79]
89.	Quinic acid	C_7_H_12_O_6_	192.1666	191		111; 173	[20,28,57,59]
90.	Dihydroferulic acid	C_10_H_12_O_4_	196.1999	195		159; 129; 113; 122	[28,80,81]
91.	Ethyl gallate	C_9_H_10_O_5_	198.1727	197		169; 125	[45]
92.	L-Tryptophan [tryptophan; (S)-tryptophan]	C_11_H_12_N_2_O_2_	204.2252		205	188; 146; 170; 118	[41,66]
93.	Myristoleic acid [cis-9-tetradecanoic acid]	C_14_H_26_O_2_	226.3550		227	209; 181; 155; 199; 181; 127	[28]
94.	Resveratrol [trans-resveratrol; stilbentriol]	C_14_H_12_O_3_	228.2433		229	142; 184; 114	[28,43]
95.	Linolenic acid (alpha-linolenic acid; linolenate)	C_18_H_30_O_2_	278.4296		279	260; 176; 120	[62,74]
96.	9-oxo-10E,12Z-octadecanoic acid [9-oxo-ODE]	C_18_H_30_O_3_	294.4290		295	249; 165; 220; 125	[62,82]
97.	Nonadecadienoic acid	C_19_H_34_O_2_	294.4721		295	278; 250; 211; 172; 204; 181; 176	[28]
98.	Protocatechuic acid-*O*-hexoside	C_13_H_16_O_9_	316.2607	315		153; 298; 151	[57,69,75]
99.	Bilobalide [(-)-Bilobalide]	C_15_H_18_O_8_	326.2986	325		183; 261; 119; 183	[46,50,75]
100.	3,7-dimethylquercetin	C_17_H_14_O_7_	330.2889		331	314; 297; 255; 228; 203; 146; 267; 227; 203; 186; 164; 134	[75]
101.	Galloyl glucose [beta-glucogallin; 1-*O*-galloyl-beta-D-glucose]	C_13_H_16_O_10_	332.2601	331		313; 195; 166	[41]
102.	Gallic acid hexoside	C_13_H_16_O_10_	332.2601	331		271; 169; 125	[83]
103.	Erucic acid (cis-13-docosenoic acid)	C_22_H_42_O_2_	338.5677		339	132; 293	[65]
104.	Esculin [aesculin; esculoside; polichrome]	C_15_H_16_O_9_	340.2821	339		177; 293; 131	[20,28,56]
105.	Palmatine [berbericinine; Burasaine]	C_21_H_22_NO_4_	352.4037		353	335; 235; 317; 235; 137	[84]
106.	Hexose-hexose-N-acetyl	C_14_H_25_NO_10_	367.3490	366		186; 142	[85]
107.	Fraxin (fraxetin-8-*O*-glucoside)	C_16_H_18_O_10_	370.3081		371	208; 352; 135	[20]
108.	1-*O*-sinapoyl-beta-D-glucose	C_17_H_22_O_10_	386.3576		387	205; 130	[20]
109.	Polydatin [piceid; trans-piceid]	C_20_H_22_O_8_	390.3839	389		227; 343; 184; 143	[27,43]
110.	Fucosterol [fucostein; trans-24-ethylidenecholesterol]	C_29_H_48_O	412.6908		413	395; 355; 271; 194; 119; 297; 199; 268; 187	[28]
111.	Stigmasterol [stigmasterin; beta-stigmasterol]	C_29_H_48_O	412.6908		413	301; 259; 189; 171	[28,86,87]
112.	Phlorizin [phloridzin; phlorizoside; floridzin: phlorrhizin; phloretin 2’-glucoside; phloretin-*O*-hexoside]	C_21_H_24_O_10_	436.4093		437	397; 217; 377	[20,27,46,49,57]
113.	Oleanoic acid	C_30_H_48_O_3_	456.7003		457	439; 411; 365; 337; 293; 248; 205; 364; 309; 219; 319; 301; 279; 247; 232	[24,76]
114.	Ursolic acid	C_30_H_48_O_3_	456.7003		457	411; 393; 365; 337; 279; 247; 292; 247; 219; 205	[63,76,86]
115.	Anmurcoic acid	C_30_H_46_O_5_	486.6922		487	469; 427; 397; 367; 325; 307; 304; 261; 279	[76]
116.	Dimethylellagic acid hexose	C_22_H_20_O_13_	492.3864		493	331; 299; 270; 242; 179; 150; 225	[41]
117.	Procyanidin A-type dimer	C_30_H_24_O_12_	576.501		577	425; 397; 373; 287; 245; 181; 245; 218; 189; 123	[20,55,57]
118.	Cyclopassifloic acid glucoside	C_37_H_62_O_12_	698.8810		699	537; 347; 271; 259; 185	[66]
